# Supplementary material for: MetaRibo-Seq measures translation in microbiomes
Source: Nat Commun. 2020 Jun 29;11:3268. doi: 10.1038/s41467-020-17081-z (PMC7324362; doi:10.1038/s41467-020-17081-z)
Supplement: Supplementary file 10 — Supplementary Data 7 [file 41467_2020_17081_MOESM10_ESM.zip › File2/Confidence_High_Taxonomy/306063_out.krona.html]

Javascript must be enabled to view this page.

members
magnitude
magnitudeUnassigned
count
unassigned
taxon
rank

306063\_out

243

243
superkingdom
2

243
phylum
976

243
200643
class

171549
order
243

2005525
family
213

375288
genus
213

1750560
species
2

SRS016132\_contig\_number\_210SRS143780\_contig\_number\_5081

211

SRS012969\_contig\_number\_33173SRS013098\_contig\_number\_12636SRS013476\_contig\_number\_33274SRS013687\_contig\_number\_34129SRS013800\_contig\_number\_31SRS013940\_contig\_number\_2689SRS013951\_contig\_number\_35574SRS014235\_contig\_number\_39916SRS014287\_contig\_number\_19123SRS014412\_contig\_number\_17756SRS014415\_contig\_number\_442SRS014459\_contig\_number\_39860SRS014613\_contig\_number\_2752SRS014613\_contig\_number\_21438SRS014683\_contig\_number\_14906SRS014923\_contig\_number\_39978SRS014948\_contig\_number\_20020SRS015065\_contig\_number\_2623SRS015217\_contig\_number\_21375SRS015264\_contig\_number\_16759SRS015579\_contig\_number\_contig-100\_775.141843SRS015663\_contig\_number\_13599SRS015694\_contig\_number\_24098SRS015782\_contig\_number\_45943SRS016056\_contig\_number\_411SRS016381\_contig\_number\_280SRS016541\_contig\_number\_15429SRS016753\_contig\_number\_2826SRS016840\_contig\_number\_2388SRS017247\_contig\_number\_976SRS017307\_contig\_number\_4338SRS017433\_contig\_number\_14364SRS017521\_contig\_number\_51SRS017701\_contig\_number\_237SRS017859\_contig\_number\_557SRS018049\_contig\_number\_30SRS018133\_contig\_number\_6148SRS018836\_contig\_number\_4235SRS018888\_contig\_number\_contig-100\_810.50366SRS018936\_contig\_number\_1197SRS019030\_contig\_number\_3447SRS019068\_contig\_number\_118862SRS019178\_contig\_number\_4720SRS019381\_contig\_number\_536SRS019397\_contig\_number\_438SRS019445\_contig\_number\_20213SRS019685\_contig\_number\_30136SRS019808\_contig\_number\_19664SRS019910\_contig\_number\_23780SRS019968\_contig\_number\_3307SRS020271\_contig\_number\_3761SRS020328\_contig\_number\_24243SRS022137\_contig\_number\_10846SRS022524\_contig\_number\_11508SRS023526\_contig\_number\_28128SRS023583\_contig\_number\_5350SRS024009\_contig\_number\_1364SRS024435\_contig\_number\_12408SRS024492\_contig\_number\_7796SRS042966\_contig\_number\_2751SRS043841\_contig\_number\_10537SRS045004\_contig\_number\_26922SRS045244\_contig\_number\_3670SRS045645\_contig\_number\_600SRS045713\_contig\_number\_contig-100\_1229.124459SRS045739\_contig\_number\_446SRS046068\_contig\_number\_135SRS046369\_contig\_number\_13450SRS046717\_contig\_number\_8150SRS047044\_contig\_number\_13714SRS048164\_contig\_number\_29914SRS048870\_contig\_number\_3355SRS048981\_contig\_number\_489SRS049402\_contig\_number\_867SRS049712\_contig\_number\_22564SRS049773\_contig\_number\_28910SRS049896\_contig\_number\_13941SRS049959\_contig\_number\_41087SRS049995\_contig\_number\_16923SRS050026\_contig\_number\_2720SRS050299\_contig\_number\_3293SRS050422\_contig\_number\_22617SRS050422\_contig\_number\_contig-100\_732.175831SRS050520\_contig\_number\_contig-100\_316.23757SRS050925\_contig\_number\_377SRS050941\_contig\_number\_21437SRS051610\_contig\_number\_contig-100\_1779.67249SRS051882\_contig\_number\_17090SRS052078\_contig\_number\_440SRS052576\_contig\_number\_324SRS052697\_contig\_number\_32511SRS053356\_contig\_number\_36435SRS053398\_contig\_number\_3181SRS053649\_contig\_number\_3901SRS054059\_contig\_number\_2805SRS054352\_contig\_number\_2146SRS054905\_contig\_number\_contig-100\_1048.121895SRS058070\_contig\_number\_11702SRS058770\_contig\_number\_15887SRS062427\_contig\_number\_8159SRS062654\_contig\_number\_24742SRS063040\_contig\_number\_8148SRS063127\_contig\_number\_1361SRS063190\_contig\_number\_9998SRS063985\_contig\_number\_2533SRS064232\_contig\_number\_874SRS064276\_contig\_number\_3808SRS064557\_contig\_number\_9664SRS065176\_contig\_number\_14178SRS065504\_contig\_number\_36368SRS074670\_contig\_number\_contig-100\_923.62577SRS075021\_contig\_number\_1315SRS075341\_contig\_number\_19417SRS075773\_contig\_number\_34221SRS075821\_contig\_number\_2908SRS075984\_contig\_number\_23184SRS076804\_contig\_number\_347SRS076876\_contig\_number\_411SRS076976\_contig\_number\_8801SRS077086\_contig\_number\_6340SRS077231\_contig\_number\_18930SRS077294\_contig\_number\_16640SRS077454\_contig\_number\_9909SRS077552\_contig\_number\_3298SRS077589\_contig\_number\_contig-100\_66.53474SRS077641\_contig\_number\_20830SRS078176\_contig\_number\_contig-100\_686.138747SRS078242\_contig\_number\_22042SRS078419\_contig\_number\_21152SRS097889\_contig\_number\_4736SRS097920\_contig\_number\_contig-100\_1125.190368SRS097958\_contig\_number\_18447SRS098061\_contig\_number\_1327SRS098571\_contig\_number\_36286SRS098644\_contig\_number\_49483SRS100907\_contig\_number\_474SRS101376\_contig\_number\_contig-100\_892.159370SRS101433\_contig\_number\_7397SRS103987\_contig\_number\_27662SRS104036\_contig\_number\_8255SRS1041039\_contig\_number\_24594SRS1041091\_contig\_number\_12544SRS1041091\_contig\_number\_contig-100\_15.95605SRS1041092\_contig\_number\_667SRS1041112\_contig\_number\_1952SRS1041116\_contig\_number\_1672SRS1041118\_contig\_number\_2297SRS1041134\_contig\_number\_8978SRS1041136\_contig\_number\_contig-100\_17.128344SRS1041137\_contig\_number\_754SRS1041138\_contig\_number\_878SRS1041139\_contig\_number\_906SRS1041140\_contig\_number\_21879SRS1041141\_contig\_number\_600SRS104311\_contig\_number\_23716SRS104327\_contig\_number\_6162SRS104636\_contig\_number\_4635SRS104912\_contig\_number\_14828SRS104975\_contig\_number\_14354SRS105082\_contig\_number\_8782SRS1054716\_contig\_number\_1095SRS1055069\_contig\_number\_4415SRS140513\_contig\_number\_contig-100\_95.98873SRS142503\_contig\_number\_49954SRS142542\_contig\_number\_21386SRS142890\_contig\_number\_32774SRS142923\_contig\_number\_3615SRS142923\_contig\_number\_16102SRS142980\_contig\_number\_2538SRS143070\_contig\_number\_contig-100\_664.117239SRS143181\_contig\_number\_4886SRS143181\_contig\_number\_contig-100\_1526.78862SRS143722\_contig\_number\_9946SRS143780\_contig\_number\_6064SRS143876\_contig\_number\_11526SRS143991\_contig\_number\_15650SRS144297\_contig\_number\_2795SRS144506\_contig\_number\_4159SRS144537\_contig\_number\_39224SRS144603\_contig\_number\_1949SRS144714\_contig\_number\_29592SRS145308\_contig\_number\_473SRS145497\_contig\_number\_contig-100\_1528.114679SRS146764\_contig\_number\_33482SRS146813\_contig\_number\_26681SRS147139\_contig\_number\_29908SRS147346\_contig\_number\_43917SRS147425\_contig\_number\_3698SRS147557\_contig\_number\_9750SRS147653\_contig\_number\_5244SRS148091\_contig\_number\_1786SRS148159\_contig\_number\_18629SRS148196\_contig\_number\_39896SRS148253\_contig\_number\_10483SRS148424\_contig\_number\_33772SRS148721\_contig\_number\_36277SRS148784\_contig\_number\_5518SRS148817\_contig\_number\_7581SRS149075\_contig\_number\_11299SRS149181\_contig\_number\_12995SRS893170\_contig\_number\_7819SRS893230\_contig\_number\_3325SRS893252\_contig\_number\_16792SRS893292\_contig\_number\_290SRS893292\_contig\_number\_8991SRS893295\_contig\_number\_43SRS893300\_contig\_number\_2264SRS893327\_contig\_number\_7739SRS893378\_contig\_number\_6466SRS893382\_contig\_number\_885SRS893383\_contig\_number\_22599
species
46503

171550
family
3

3
genus
239759

2

SRS042628\_contig\_number\_29881SRS078665\_contig\_number\_6370
28117
species

1

SRS148319\_contig\_number\_537
214856
species

1853231
family
2

574697
genus
1

1472416
species

SRS098644\_contig\_number\_11398
1

1
283168
genus


SRS019161\_contig\_number\_24864
1
28118
species

family
815
21

genus
3
21

SRS024132\_contig\_number\_9206SRS101376\_contig\_number\_3949SRS1041130\_contig\_number\_4837
816

1

SRS1041136\_contig\_number\_17984
817
species

818
species
2

SRS020233\_contig\_number\_42272SRS893358\_contig\_number\_8937

species
2292284

SRS143148\_contig\_number\_6015
1

species
371601

SRS011405\_contig\_number\_341SRS044535\_contig\_number\_14267
2


SRS077849\_contig\_number\_28048
1
species
2292944

357276
species

SRS019286\_contig\_number\_422
1


SRS024075\_contig\_number\_10207SRS140492\_contig\_number\_25846SRS140513\_contig\_number\_851
3
820
species

821
species
2

SRS063489\_contig\_number\_3577SRS143991\_contig\_number\_27619

46506
species
2

SRS017821\_contig\_number\_2569SRS047433\_contig\_number\_10791

species
28116
2

SRS143372\_contig\_number\_1775SRS147557\_contig\_number\_23121

1

SRS055966\_contig\_number\_1122
species
47678

family
171552
4

3
838
genus

165179
species
2

SRS023914\_contig\_number\_12674SRS064757\_contig\_number\_614

1262933
species

SRS051031\_contig\_number\_841
1

577309
genus
1

454154
species

SRS013521\_contig\_number\_719
1
